# TP53 p.Arg337His in Brazilian lung cancer: when tumour profiling should trigger germline evaluation

**DOI:** 10.1016/j.lana.2026.101569

**Published:** 2026-07-13

**Authors:** Renata Lazari Sandoval

**Affiliations:** Oncology Center, Hospital Sírio-Libanês, Brasília, Federal District, Brazil

Cavagna and colleagues provide one of the largest real-world molecular characterisations of lung cancer in Brazil, reinforcing the clinical relevance of broad tumour profiling and *TP53* co-mutations in *EGFR*-mutant disease.^1^ We highlight an additional implication of their supplementary data.

In Supplementary Table 1, *TP53* p.Arg337His appears in 36 tumour entries, corresponding to 3.2% of the cohort and 5.5% of *TP53*-mutated tumours. Although *TP53* is frequently altered in lung cancer, p.Arg337His is not a usual recurrent somatic hotspot. It is a Brazilian founder pathogenic variant associated with Li-Fraumeni syndrome. In our Brazilian pan-cancer tumour profiling cohort, p.Arg337His was found in 15 of 755 tumours, including eight lung cancers; all tested patients carried germline *TP53* p.Arg337His, and most did not meet clinical criteria.^2^

Lung cancer is not a classic core Li-Fraumeni tumour, but Brazilian data support lung adenocarcinoma as part of the expanded *TP53* p.Arg337His spectrum, often with *EGFR* or *HER*-family alterations.^3^, ^4^, ^5^ These findings do not yet justify universal p.R337H genotyping in all Brazilian lung adenocarcinomas, given regional variability and the need for cost-effectiveness analyses. However, Brazilian patients with *EGFR*-mutated lung adenocarcinoma may be a priority group for prospective targeted p.Arg337His testing.

Detection of *TP53* p.Arg337His in tumour-only sequencing should prompt genetic counselling and confirmatory germline testing. Exploratory comparisons with other *TP53*-mutated adenocarcinomas, or sensitivity analyses excluding p.Arg337His from the broader *TP53*-mutated group, could clarify whether this founder-enriched subset differs clinically or prognostically.

## Contributors

RLS conceived the correspondence, reviewed and interpreted the cited literature and supplementary data, drafted and revised the manuscript, approved the final submitted version, and agrees to be accountable for all aspects of the work.

## Declaration of interests

RLS is an author of references 2 and 5.
